# Supplementary material for: Mechanisms governing the pioneering and redistribution capabilities of the non-classical pioneer PU.1
Source: Nat Commun. 2020 Jan 21;11:402. doi: 10.1038/s41467-019-13960-2 (PMC6972792; doi:10.1038/s41467-019-13960-2)
Supplement: Supplementary file 7 — Source data [file 41467_2019_13960_MOESM7_ESM.zip › Source_Data/FigureS4/FigureS4G_motifAnnotation/bound.pairs.PU1notmasked/knownResults.html]

/misc/data/analysis/project\_PU1/CTV1/motifs/basic/PU1.ntag15.filtered.12-50-bp.cluster.PU1notmasked - Homer Known Motif Enrichment Results


# Homer Known Motif Enrichment Results (/misc/data/analysis/project\_PU1/CTV1/motifs/basic/PU1.ntag15.filtered.12-50-bp.cluster.PU1notmasked)

Homer *de novo* Motif Results  
Gene Ontology Enrichment Results  
Known Motif Enrichment Results (txt file)  
Total Target Sequences = 10146, Total Background Sequences = 34174

|  |  |  |  |  |  |  |  |  |  |  |  |
| --- | --- | --- | --- | --- | --- | --- | --- | --- | --- | --- | --- |
| Rank | Motif | Name | P-value | log P-pvalue | q-value (Benjamini) | # Target Sequences with Motif | % of Targets Sequences with Motif | # Background Sequences with Motif | % of Background Sequences with Motif | Motif File | SVG |
| 1 | C G T A T A C G T C G A A C T G A C T G C G T A C G T A T A C G A G C T T A C G | PU.1(ETS)/ThioMac-PU.1-ChIP-Seq(GSE21512)/Homer | 1e-3177 | -7.317e+03 | 0.0000 | 6589.0 | 64.94% | 2221.3 | 6.50% | motif file (matrix) | svg |
| 2 | C G T A C T G A C G T A C T A G T C G A C T A G A C T G C G T A C G T A T A C G A G C T A T C G | SpiB(ETS)/OCILY3-SPIB-ChIP-Seq(GSE56857)/Homer | 1e-2552 | -5.879e+03 | 0.0000 | 5071.0 | 49.98% | 1177.0 | 3.44% | motif file (matrix) | svg |
| 3 | T C G A T A G C T G C A A C T G A C T G C G T A C G T A C T A G G A C T T A C G | ETS1(ETS)/Jurkat-ETS1-ChIP-Seq(GSE17954)/Homer | 1e-2250 | -5.183e+03 | 0.0000 | 6454.0 | 63.61% | 4053.7 | 11.86% | motif file (matrix) | svg |
| 4 | C G T A T A G C T A G C T G C A A C T G C T A G C G T A C G T A T C A G G A C T | EHF(ETS)/LoVo-EHF-ChIP-Seq(GSE49402)/Homer | 1e-2233 | -5.142e+03 | 0.0000 | 7187.0 | 70.83% | 5773.7 | 16.89% | motif file (matrix) | svg |
| 5 | G C T A A G T C T A C G T G C A A T C G T C A G G C T A T C G A T C A G A G C T | ELF5(ETS)/T47D-ELF5-ChIP-Seq(GSE30407)/Homer | 1e-2061 | -4.747e+03 | 0.0000 | 5694.0 | 56.12% | 3085.4 | 9.03% | motif file (matrix) | svg |
| 6 | T C G A C T G A T A G C T G A C T C A G T C A G C G T A C G T A T C A G A G C T | ETV1(ETS)/GIST48-ETV1-ChIP-Seq(GSE22441)/Homer | 1e-1643 | -3.784e+03 | 0.0000 | 6085.0 | 59.97% | 5144.1 | 15.05% | motif file (matrix) | svg |
| 7 | A T G C A G T C C T G A A G T C C G A T A C G T A G T C A G T C A C G T A T C G G A C T A C G T | Etv2(ETS)/ES-ER71-ChIP-Seq(GSE59402)/Homer(0.967) | 1e-1569 | -3.614e+03 | 0.0000 | 5316.0 | 52.39% | 3753.9 | 10.98% | motif file (matrix) | svg |
| 8 | C G T A T G A C T A G C T G C A A C T G A C T G C G T A C G T A T C A G G A C T | ELF3(ETS)/PDAC-ELF3-ChIP-Seq(GSE64557)/Homer | 1e-1540 | -3.548e+03 | 0.0000 | 4919.0 | 48.48% | 3086.1 | 9.03% | motif file (matrix) | svg |
| 9 | T C G A T A G C G T C A A C T G A C T G C G T A C G T A C T A G A G C T T C A G | ERG(ETS)/VCaP-ERG-ChIP-Seq(GSE14097)/Homer | 1e-1531 | -3.526e+03 | 0.0000 | 6538.0 | 64.43% | 6678.9 | 19.54% | motif file (matrix) | svg |
| 10 | A G T C C T G A A G T C C G A T C A G T G A T C A T G C A C T G A T C G G A C T | Fli1(ETS)/CD8-FLI-ChIP-Seq(GSE20898)/Homer | 1e-1436 | -3.309e+03 | 0.0000 | 5114.0 | 50.40% | 3777.7 | 11.05% | motif file (matrix) | svg |
| 11 | T C G A T C G A T A G C G T A C T C A G T A C G C G T A C G T A T C A G A G C T | GABPA(ETS)/Jurkat-GABPa-ChIP-Seq(GSE17954)/Homer | 1e-1362 | -3.138e+03 | 0.0000 | 4648.0 | 45.81% | 3120.7 | 9.13% | motif file (matrix) | svg |
| 12 | T G A C C T A G T C A G G T C A C G T A T C A G C G A T T C A G T C G A T G C A C T G A T A G C | PU.1-IRF(ETS:IRF)/Bcell-PU.1-ChIP-Seq(GSE21512)/Homer | 1e-841 | -1.939e+03 | 0.0000 | 5485.0 | 54.06% | 7219.8 | 21.12% | motif file (matrix) | svg |
| 13 | T C G A A G C T A C G T A C G T A G T C A G T C A C G T A T C G G A C T A T C G | EWS:ERG-fusion(ETS)/CADO\_ES1-EWS:ERG-ChIP-Seq(SRA014231)/Homer | 1e-769 | -1.771e+03 | 0.0000 | 3584.0 | 35.32% | 3181.9 | 9.31% | motif file (matrix) | svg |
| 14 | T G C A C T G A A T G C G T C A A C T G A C T G C G T A C G T A C T A G A G C T | Ets1-distal(ETS)/CD4+-PolII-ChIP-Seq(Barski\_et\_al.)/Homer | 1e-705 | -1.624e+03 | 0.0000 | 2266.0 | 22.33% | 1146.8 | 3.35% | motif file (matrix) | svg |
| 15 | C T G A T G C A T A G C T G A C T A C G T C A G C T G A G C T A T C A G G A C T | ELF1(ETS)/Jurkat-ELF1-ChIP-Seq(SRA014231)/Homer | 1e-639 | -1.472e+03 | 0.0000 | 2250.0 | 22.17% | 1306.9 | 3.82% | motif file (matrix) | svg |
| 16 | T G C A T C G A T A G C G T A C T C A G C T A G G T C A G C T A T C A G G A C T | ETS(ETS)/Promoter/Homer | 1e-502 | -1.156e+03 | 0.0000 | 1652.0 | 16.28% | 829.4 | 2.43% | motif file (matrix) | svg |
| 17 | T C G A T A G C G T C A A C T G C T A G C G T A C G A T A C T G A C G T A C T G A C T G A C G T | ETS:RUNX(ETS,Runt)/Jurkat-RUNX1-ChIP-Seq(GSE17954)/Homer | 1e-320 | -7.374e+02 | 0.0000 | 883.0 | 8.70% | 301.0 | 0.88% | motif file (matrix) | svg |
| 18 | G A T C T C G A A G T C C G A T C G A T A G T C A T G C A C T G A T C G G A C T | Elk1(ETS)/Hela-Elk1-ChIP-Seq(GSE31477)/Homer | 1e-269 | -6.205e+02 | 0.0000 | 1450.0 | 14.29% | 1308.3 | 3.83% | motif file (matrix) | svg |
| 19 | T G C A C T G A A G T C G T C A A C T G A C T G C G T A C G T A C T G A A G C T | EWS:FLI1-fusion(ETS)/SK\_N\_MC-EWS:FLI1-ChIP-Seq(SRA014231)/Homer | 1e-263 | -6.061e+02 | 0.0000 | 1781.0 | 17.55% | 1975.0 | 5.78% | motif file (matrix) | svg |
| 20 | C T G A A T G C C G T A A C G T A G T C A G T C A C G T A C T G A T C G G C A T | SPDEF(ETS)/VCaP-SPDEF-ChIP-Seq(SRA014231)/Homer | 1e-244 | -5.635e+02 | 0.0000 | 2469.0 | 24.33% | 3617.5 | 10.58% | motif file (matrix) | svg |
| 21 | C T A G C T A G C G T A C G T A T A C G C G A T C T A G C T G A C T G A C G T A T A C G G A C T | PU.1:IRF8(ETS:IRF)/pDC-Irf8-ChIP-Seq(GSE66899)/Homer | 1e-243 | -5.602e+02 | 0.0000 | 1163.0 | 11.46% | 920.6 | 2.69% | motif file (matrix) | svg |
| 22 | T C A G T C A G G C T A C G T A T A C G G A C T T C A G T C G A C T G A C G T A T A C G G A C T | IRF8(IRF)/BMDM-IRF8-ChIP-Seq(GSE77884)/Homer | 1e-207 | -4.780e+02 | 0.0000 | 1416.0 | 13.95% | 1554.2 | 4.55% | motif file (matrix) | svg |
| 23 | G A T C C T G A A G T C C G A T C G A T G A T C A G T C A C T G A T C G A G C T | Elk4(ETS)/Hela-Elk4-ChIP-Seq(GSE31477)/Homer | 1e-140 | -3.231e+02 | 0.0000 | 1061.0 | 10.46% | 1237.4 | 3.62% | motif file (matrix) | svg |
| 24 | C A G T A C T G T C A G T G C A G C T A A T G C T C G A A T C G G T C A T G C A | ZNF189(Zf)/HEK293-ZNF189.GFP-ChIP-Seq(GSE58341)/Homer | 1e-78 | -1.817e+02 | 0.0000 | 1731.0 | 17.06% | 3404.5 | 9.96% | motif file (matrix) | svg |
| 25 | G A C T C T A G C T A G C T A G A C T G T C G A C T G A C T A G C T A G C T A G G T A C G T C A | ZNF467(Zf)/HEK293-ZNF467.GFP-ChIP-Seq(GSE58341)/Homer | 1e-77 | -1.785e+02 | 0.0000 | 1746.0 | 17.21% | 3463.8 | 10.13% | motif file (matrix) | svg |
| 26 | T C G A T C G A T A C G G A T C G T C A G T A C C G A T A G C T G T C A T G C A | Nkx3.1(Homeobox)/LNCaP-Nkx3.1-ChIP-Seq(GSE28264)/Homer | 1e-20 | -4.694e+01 | 0.0000 | 3094.0 | 30.49% | 8804.7 | 25.76% | motif file (matrix) | svg |
| 27 | A G T C A C G T A C T G A G C T A C G T A C G T G T C A A G T C | Foxo1(Forkhead)/RAW-Foxo1-ChIP-Seq(Fan\_et\_al.)/Homer | 1e-18 | -4.226e+01 | 0.0000 | 2380.0 | 23.46% | 6623.3 | 19.38% | motif file (matrix) | svg |
| 28 | T C G A A C T G A C T G C G T A C G T A T C G A A G T C C T G A A T C G G T A C G C A T C A T G | ETS:E-box(ETS,bHLH)/HPC7-Scl-ChIP-Seq(GSE22178)/Homer | 1e-17 | -4.129e+01 | 0.0000 | 198.0 | 1.95% | 289.5 | 0.85% | motif file (matrix) | svg |
| 29 | G C T A C T G A T C G A A G T C A G T C C T G A A G T C G T C A C T G A T G C A | RUNX1(Runt)/Jurkat-RUNX1-ChIP-Seq(GSE29180)/Homer | 1e-17 | -3.960e+01 | 0.0000 | 1170.0 | 11.53% | 2958.3 | 8.65% | motif file (matrix) | svg |
| 30 | T A G C G C T A T C G A C T G A A G T C A G T C C T G A A G T C C G T A C T A G | RUNX(Runt)/HPC7-Runx1-ChIP-Seq(GSE22178)/Homer | 1e-14 | -3.370e+01 | 0.0000 | 734.0 | 7.23% | 1754.4 | 5.13% | motif file (matrix) | svg |
| 31 | C T G A T A C G G C A T A G C T A G C T A G T C T C G A A C T G C A G T A G C T A G C T G A T C | IRF3(IRF)/BMDM-Irf3-ChIP-Seq(GSE67343)/Homer | 1e-14 | -3.361e+01 | 0.0000 | 587.0 | 5.78% | 1339.5 | 3.92% | motif file (matrix) | svg |
| 32 | C T A G C T A G A G T C T C A G A C T G A C G T A C G T C T G A | MYB(HTH)/ERMYB-Myb-ChIPSeq(GSE22095)/Homer | 1e-13 | -3.179e+01 | 0.0000 | 1751.0 | 17.26% | 4838.9 | 14.16% | motif file (matrix) | svg |
| 33 | T C G A G A C T A G C T T G A C A G C T G T A C T C A G G A T C A T C G T G C A A C T G C T G A | GFX(?)/Promoter/Homer | 1e-13 | -3.160e+01 | 0.0000 | 44.0 | 0.43% | 21.9 | 0.06% | motif file (matrix) | svg |
| 34 | T G C A T A G C G A C T T G C A T G A C T G C A C G T A A G C T A G C T A G T C A G T C G T A C | GFY(?)/Promoter/Homer | 1e-12 | -2.936e+01 | 0.0000 | 125.0 | 1.23% | 173.3 | 0.51% | motif file (matrix) | svg |
| 35 | T A C G T C A G T G C A A G C T T G A C A G C T A G T C A C T G G A T C A C T G T C G A A C T G C T G A C T G A A T G C | ZBTB33(Zf)/GM12878-ZBTB33-ChIP-Seq(GSE32465)/Homer | 1e-12 | -2.824e+01 | 0.0000 | 59.0 | 0.58% | 48.8 | 0.14% | motif file (matrix) | svg |
| 36 | A G C T C T G A C T A G C T A G A C T G T A G C T G C A T C G A C T G A C T A G C A T G A C G T A T G C T C G A | RXR(NR),DR1/3T3L1-RXR-ChIP-Seq(GSE13511)/Homer | 1e-12 | -2.778e+01 | 0.0000 | 1586.0 | 15.63% | 4396.9 | 12.86% | motif file (matrix) | svg |
| 37 | G A C T C T A G C T A G A G T C T G C A A C T G A C G T A C G T C T A G T C A G | AMYB(HTH)/Testes-AMYB-ChIP-Seq(GSE44588)/Homer | 1e-10 | -2.385e+01 | 0.0000 | 1521.0 | 14.99% | 4266.6 | 12.48% | motif file (matrix) | svg |
| 38 | T G A C G C T A T C G A T G C A A G T C A G T C C G T A A G T C C G T A C T G A G C T A G T A C | RUNX2(Runt)/PCa-RUNX2-ChIP-Seq(GSE33889)/Homer | 1e-9 | -2.201e+01 | 0.0000 | 859.0 | 8.47% | 2266.2 | 6.63% | motif file (matrix) | svg |
| 39 | C T G A T C A G G T A C G C T A A C T G T G A C G C A T C A T G | SCL(bHLH)/HPC7-Scl-ChIP-Seq(GSE13511)/Homer | 1e-9 | -2.133e+01 | 0.0000 | 4461.0 | 43.96% | 13864.5 | 40.56% | motif file (matrix) | svg |
| 40 | T G C A A G C T C T G A A T C G G A C T C T A G G T A C G A T C G T C A A G T C G T A C G A C T C T A G A T C G G C A T C A T G C A T G G A T C G T A C C T G A | CTCF(Zf)/CD4+-CTCF-ChIP-Seq(Barski\_et\_al.)/Homer | 1e-8 | -2.000e+01 | 0.0000 | 120.0 | 1.18% | 199.8 | 0.58% | motif file (matrix) | svg |
| 41 | T C A G C T G A C G T A C G T A T A C G G C A T C T A G C T G A C G T A C G T A T A C G G A C T | IRF1(IRF)/PBMC-IRF1-ChIP-Seq(GSE43036)/Homer | 1e-8 | -1.975e+01 | 0.0000 | 327.0 | 3.22% | 742.6 | 2.17% | motif file (matrix) | svg |
| 42 | A C T G G A T C G A C T A C T G A C G T C A T G A C T G A C G T A G C T C G A T | RUNX-AML(Runt)/CD4+-PolII-ChIP-Seq(Barski\_et\_al.)/Homer | 1e-8 | -1.882e+01 | 0.0000 | 745.0 | 7.34% | 1971.4 | 5.77% | motif file (matrix) | svg |
| 43 | C A T G A C T G C T A G T C G A T C G A T C G A T C G A T C A G T C A G T C A G T G A C T G A C C G T A A C T G T G C A C G A T A C T G | RBPJ:Ebox(?,bHLH)/Panc1-Rbpj1-ChIP-Seq(GSE47459)/Homer | 1e-7 | -1.814e+01 | 0.0000 | 514.0 | 5.07% | 1294.7 | 3.79% | motif file (matrix) | svg |
| 44 | T G A C G C T A T G A C C G T A T C A G G A T C C G T A C A T G C A T G C T A G C T A G C T A G | Unknown-ESC-element(?)/mES-Nanog-ChIP-Seq(GSE11724)/Homer | 1e-7 | -1.767e+01 | 0.0000 | 656.0 | 6.46% | 1721.6 | 5.04% | motif file (matrix) | svg |
| 45 | T G A C C T G A C T A G T C G A C T G A A T G C C G T A A C T G G C A T G T A C G C A T A T C G G C A T A G C T G A T C | PR(NR)/T47D-PR-ChIP-Seq(GSE31130)/Homer | 1e-7 | -1.766e+01 | 0.0000 | 2427.0 | 23.92% | 7292.0 | 21.33% | motif file (matrix) | svg |
| 46 | T C G A T G A C G C A T A G C T C A G T G A T C G C T A G A T C G A C T A C G T G C A T A G T C | PRDM1(Zf)/Hela-PRDM1-ChIP-Seq(GSE31477)/Homer | 1e-7 | -1.747e+01 | 0.0000 | 1061.0 | 10.46% | 2959.1 | 8.66% | motif file (matrix) | svg |
| 47 | C T G A C G A T C T A G T C A G G A T C C T G A T C A G G A T C C T G A A C T G A G T C G C T A A C G T A G T C G C A T | PRDM9(Zf)/Testis-DMC1-ChIP-Seq(GSE35498)/Homer | 1e-7 | -1.726e+01 | 0.0000 | 463.0 | 4.56% | 1156.8 | 3.38% | motif file (matrix) | svg |
| 48 | A C T G C T A G G A T C C G T A C T A G A G T C T G C A G A C T C G T A A C G T T C A G A G T C A C G T C T G A A G T C A G T C G A T C C G T A T C A G T A C G | EBNA1(EBV-virus)/Raji-EBNA1-ChIP-Seq(GSE30709)/Homer | 1e-6 | -1.387e+01 | 0.0000 | 34.0 | 0.34% | 34.3 | 0.10% | motif file (matrix) | svg |
| 49 | T C A G A G C T A T G C C G T A A G C T T C A G C A G T A C T G C T G A A G T C | MITF(bHLH)/MastCells-MITF-ChIP-Seq(GSE48085)/Homer | 1e-5 | -1.365e+01 | 0.0000 | 934.0 | 9.20% | 2641.3 | 7.73% | motif file (matrix) | svg |
| 50 | T A C G T A C G G T A C A T C G A C T G T A C G T C G A C T G A T C G A A T C G | E2F6(E2F)/Hela-E2F6-ChIP-Seq(GSE31477)/Homer | 1e-5 | -1.330e+01 | 0.0000 | 531.0 | 5.23% | 1412.6 | 4.13% | motif file (matrix) | svg |
| 51 | C T A G T C G A G C A T C A T G G C T A T A G C C G A T G T A C C T G A A G C T | JunB(bZIP)/DendriticCells-Junb-ChIP-Seq(GSE36099)/Homer | 1e-5 | -1.290e+01 | 0.0000 | 565.0 | 5.57% | 1521.1 | 4.45% | motif file (matrix) | svg |
| 52 | C T G A T C A G A G T C C G T A A T C G A T G C C G A T A C T G A G T C G A C T A T C G A G T C | MyoD(bHLH)/Myotube-MyoD-ChIP-Seq(GSE21614)/Homer | 1e-5 | -1.287e+01 | 0.0000 | 632.0 | 6.23% | 1725.6 | 5.05% | motif file (matrix) | svg |
| 53 | C A G T T G C A A C G T A C T G C G T A A T C G C G A T T G A C C G T A A C G T | BATF(bZIP)/Th17-BATF-ChIP-Seq(GSE39756)/Homer | 1e-5 | -1.218e+01 | 0.0000 | 674.0 | 6.64% | 1866.8 | 5.46% | motif file (matrix) | svg |
| 54 | C T A G T C G A A C G T A C T G C G T A A T G C A C G T G T A C C G T A A G C T G A T C G T A C | Atf3(bZIP)/GBM-ATF3-ChIP-Seq(GSE33912)/Homer | 1e-5 | -1.216e+01 | 0.0000 | 694.0 | 6.84% | 1928.9 | 5.64% | motif file (matrix) | svg |
| 55 | C T A G C A G T T G A C C G T A G A T C T C A G G A C T C A T G | BMAL1(bHLH)/Liver-Bmal1-ChIP-Seq(GSE39860)/Homer | 1e-4 | -1.137e+01 | 0.0001 | 1692.0 | 16.67% | 5104.1 | 14.93% | motif file (matrix) | svg |
| 56 | C T G A T C A G C A G T C T A G A C T G C T A G G A T C A T C G A C T G C T G A T C A G G A T C | Sp5(Zf)/mES-Sp5.Flag-ChIP-Seq(GSE72989)/Homer | 1e-4 | -1.095e+01 | 0.0001 | 1018.0 | 10.03% | 2965.9 | 8.68% | motif file (matrix) | svg |
| 57 | T G C A C G T A A C T G T C A G C A G T C A T G T C A G G A T C T A C G A G T C T G C A A C T G A C T G T G A C G T C A | ZNF165(Zf)/WHIM12-ZNF165-ChIP-Seq(GSE65937)/Homer | 1e-4 | -1.085e+01 | 0.0001 | 178.0 | 1.75% | 410.7 | 1.20% | motif file (matrix) | svg |
| 58 | C G A T T A C G T G C A G T A C G A T C G A C T A G C T A C G T A T C G G T A C G A T C G T A C G A T C G T C A | PPARE(NR),DR1/3T3L1-Pparg-ChIP-Seq(GSE13511)/Homer | 1e-4 | -1.074e+01 | 0.0001 | 1254.0 | 12.36% | 3719.1 | 10.88% | motif file (matrix) | svg |
| 59 | C G T A C T A G C A T G A G C T A C T G C G A T A T C G C G T A G T C A G T C A | Tbet(T-box)/CD8-Tbet-ChIP-Seq(GSE33802)/Homer | 1e-4 | -1.066e+01 | 0.0001 | 1357.0 | 13.37% | 4050.1 | 11.85% | motif file (matrix) | svg |
| 60 | A T G C T C G A T A C G A C G T A T G C A G T C A C G T A G T C A G T C G A T C | Znf263(Zf)/K562-Znf263-ChIP-Seq(GSE31477)/Homer | 1e-4 | -1.028e+01 | 0.0002 | 2423.0 | 23.88% | 7516.2 | 21.99% | motif file (matrix) | svg |
| 61 | C G A T T G C A G T A C C G T A A G T C C T A G G A C T C A T G | NPAS(bHLH)/Liver-NPAS-ChIP-Seq(GSE39860)/Homer | 1e-4 | -1.010e+01 | 0.0002 | 1451.0 | 14.30% | 4368.5 | 12.78% | motif file (matrix) | svg |
| 62 | C T A G T C G A C T G A C G T A T A C G G A C T T C A G T C G A G T C A T G C A T A C G A G C T | IRF2(IRF)/Erythroblas-IRF2-ChIP-Seq(GSE36985)/Homer | 1e-4 | -1.003e+01 | 0.0003 | 172.0 | 1.70% | 401.1 | 1.17% | motif file (matrix) | svg |
| 63 | A G C T A C G T A C T G A T G C A G T C C G T A C T G A T A C G | NF1-halfsite(CTF)/LNCaP-NF1-ChIP-Seq(Unpublished)/Homer | 1e-4 | -9.941e+00 | 0.0003 | 1706.0 | 16.81% | 5196.0 | 15.20% | motif file (matrix) | svg |
| 64 | A T G C G A T C G A C T A G C T C G A T C G A T G T C A C G A T T C G A A T C G T A G C T A G C | TATA-Box(TBP)/Promoter/Homer | 1e-4 | -9.931e+00 | 0.0003 | 1510.0 | 14.88% | 4563.4 | 13.35% | motif file (matrix) | svg |
| 65 | A C T G C T A G T C G A C G A T C A T G G C T A A T C G C G A T G T A C G C T A A G C T G T A C | Fra1(bZIP)/BT549-Fra1-ChIP-Seq(GSE46166)/Homer | 1e-4 | -9.929e+00 | 0.0003 | 542.0 | 5.34% | 1504.0 | 4.40% | motif file (matrix) | svg |
| 66 | G A C T C T A G G A T C C A G T A C T G C T G A A T G C G C A T A T G C C T G A | MafA(bZIP)/Islet-MafA-ChIP-Seq(GSE30298)/Homer | 1e-4 | -9.682e+00 | 0.0003 | 708.0 | 6.98% | 2022.3 | 5.92% | motif file (matrix) | svg |
| 67 | T C G A C T A G A G T C A G T C C G T A C G T A A C G T T A G C T C A G T A C G | NFY(CCAAT)/Promoter/Homer | 1e-3 | -9.176e+00 | 0.0006 | 736.0 | 7.25% | 2121.8 | 6.21% | motif file (matrix) | svg |
| 68 | T C G A A C G T C A T G G C T A T A G C C G A T G T A C G C T A A C G T A T G C | AP-1(bZIP)/ThioMac-PU.1-ChIP-Seq(GSE21512)/Homer | 1e-3 | -9.102e+00 | 0.0006 | 760.0 | 7.49% | 2198.4 | 6.43% | motif file (matrix) | svg |
| 69 | A G T C G C A T C G T A C G T A G T A C A C G T A C T G G A T C G A T C T C G A | BMYB(HTH)/Hela-BMYB-ChIP-Seq(GSE27030)/Homer | 1e-3 | -8.871e+00 | 0.0007 | 1405.0 | 13.85% | 4259.3 | 12.46% | motif file (matrix) | svg |
| 70 | C A T G C T A G T C G A A C G T A C T G C G T A T A G C C G A T T G A C C G T A A G C T G A T C | Fra2(bZIP)/Striatum-Fra2-ChIP-Seq(GSE43429)/Homer | 1e-3 | -8.760e+00 | 0.0008 | 454.0 | 4.47% | 1255.2 | 3.67% | motif file (matrix) | svg |
| 71 | C T A G T C G A A C G T A C T G C G T A T A G C C G A T G T A C C G T A A G C T G A T C G T A C | Jun-AP1(bZIP)/K562-cJun-ChIP-Seq(GSE31477)/Homer | 1e-3 | -8.624e+00 | 0.0009 | 222.0 | 2.19% | 560.1 | 1.64% | motif file (matrix) | svg |
| 72 | C T A G A C T G C T A G T C A G T C A G T A C G C T A G A C T G | Maz(Zf)/HepG2-Maz-ChIP-Seq(GSE31477)/Homer | 1e-3 | -8.591e+00 | 0.0009 | 1313.0 | 12.94% | 3972.1 | 11.62% | motif file (matrix) | svg |
| 73 | T A C G T C A G A G C T A T G C C G T A A G T C T C A G A C G T A C T G T C G A | USF1(bHLH)/GM12878-Usf1-ChIP-Seq(GSE32465)/Homer | 1e-3 | -8.388e+00 | 0.0011 | 385.0 | 3.79% | 1051.1 | 3.07% | motif file (matrix) | svg |
| 74 | C T G A C T A G T G C A C T G A T C G A A G C T C A T G T C G A A G T C G A C T A C G T A G T C G A T C G A T C G A C T | ZNF528(Zf)/HEK293-ZNF528.GFP-ChIP-Seq(GSE58341)/Homer | 1e-3 | -8.354e+00 | 0.0012 | 19.0 | 0.19% | 19.8 | 0.06% | motif file (matrix) | svg |
| 75 | C T A G T C G A C G A T A C T G C G T A T A C G A G C T T G A C G C T A A C G T G A T C T A G C | Fosl2(bZIP)/3T3L1-Fosl2-ChIP-Seq(GSE56872)/Homer | 1e-3 | -7.954e+00 | 0.0017 | 305.0 | 3.01% | 816.9 | 2.39% | motif file (matrix) | svg |
| 76 | T A G C G T A C A G T C G T A C C G A T A G T C A G T C A G T C A G T C A G T C C G T A G A T C | Zfp281(Zf)/ES-Zfp281-ChIP-Seq(GSE81042)/Homer | 1e-3 | -7.951e+00 | 0.0017 | 202.0 | 1.99% | 510.3 | 1.49% | motif file (matrix) | svg |
| 77 | T C G A C T G A C T G A A T G C G T A C G T C A A G C T A G C T C G T A T C A G | Barx1(Homeobox)/Stomach-Barx1.3xFlag-ChIP-Seq(GSE69483)/Homer | 1e-3 | -7.687e+00 | 0.0022 | 544.0 | 5.36% | 1555.2 | 4.55% | motif file (matrix) | svg |
| 78 | T A G C G C T A A C T G C G T A A C G T C G T A C G T A T A C G T C A G T C G A | Gata1(Zf)/K562-GATA1-ChIP-Seq(GSE18829)/Homer | 1e-3 | -7.418e+00 | 0.0028 | 707.0 | 6.97% | 2073.3 | 6.07% | motif file (matrix) | svg |
| 79 | C G T A C G T A C G T A G C A T G C A T T A C G G T A C G A C T A C T G C G T A A T C G A C G T G T A C C G T A A G C T | Bach1(bZIP)/K562-Bach1-ChIP-Seq(GSE31477)/Homer | 1e-2 | -6.886e+00 | 0.0047 | 60.0 | 0.59% | 122.0 | 0.36% | motif file (matrix) | svg |
| 80 | A G C T A G T C A T G C A G C T A C G T C G T A A C G T A G T C C G A T A T G C | Gata2(Zf)/K562-GATA2-ChIP-Seq(GSE18829)/Homer | 1e-2 | -6.559e+00 | 0.0065 | 778.0 | 7.67% | 2322.4 | 6.79% | motif file (matrix) | svg |
| 81 | C T A G G C A T G A T C C G T A A G T C T C A G G A C T C T A G | CLOCK(bHLH)/Liver-Clock-ChIP-Seq(GSE39860)/Homer | 1e-2 | -5.978e+00 | 0.0114 | 388.0 | 3.82% | 1107.3 | 3.24% | motif file (matrix) | svg |
| 82 | G T A C C G T A C G T A T A C G G C A T G T A C C G T A C A T G A G T C C G T A C G T A C G A T G C A T G C A T G A C T | MafF(bZIP)/HepG2-MafF-ChIP-Seq(GSE31477)/Homer | 1e-2 | -5.470e+00 | 0.0187 | 290.0 | 2.86% | 814.9 | 2.38% | motif file (matrix) | svg |
| 83 | A G C T A T G C G A C T G C A T C G T A A G C T G T A C C G A T A T C G A G T C | Gata6(Zf)/HUG1N-GATA6-ChIP-Seq(GSE51936)/Homer | 1e-2 | -5.300e+00 | 0.0219 | 1054.0 | 10.39% | 3252.5 | 9.51% | motif file (matrix) | svg |
| 84 | T C G A A G T C C G T A A T C G A T G C C G A T A C T G A G T C A G C T A C T G | Tcf12(bHLH)/GM12878-Tcf12-ChIP-Seq(GSE32465)/Homer | 1e-2 | -5.145e+00 | 0.0253 | 779.0 | 7.68% | 2370.7 | 6.94% | motif file (matrix) | svg |
| 85 | C G A T T A C G G A T C G C T A T C A G G A C T C G A T G T A C G A T C G T C A T C G A T G A C C G T A C T A G G A C T C T A G C T A G G T A C A G T C C G T A | CTCF-SatelliteElement(Zf?)/CD4+-CTCF-ChIP-Seq(Barski\_et\_al.)/Homer | 1e-2 | -5.031e+00 | 0.0280 | 20.0 | 0.20% | 31.5 | 0.09% | motif file (matrix) | svg |
| 86 | C T A G T C G A C G A T C T A G G C A T C A G T C T A G G A T C C G T A G T C A | CEBP:AP1(bZIP)/ThioMac-CEBPb-ChIP-Seq(GSE21512)/Homer | 1e-2 | -4.676e+00 | 0.0394 | 727.0 | 7.16% | 2219.0 | 6.49% | motif file (matrix) | svg |
